# Overview of Randomized Controlled Trials in Primary Total Hip Arthroplasty (34,020 Patients): What Have We Learnt?

**DOI:** 10.5435/JAAOSGlobal-D-20-00120

**Published:** 2020-08-04

**Authors:** Hosam E. Matar, Simon R. Platt, Tim N. Board, Martyn L. Porter

**Affiliations:** From the Centre for Hip Surgery (Mr. Matar, Dr. Board, and Mr. Porter), Wrightington Hospital, Appley Bridge, Wigan; and the Department of Orthopaedic Surgery (Mr. Platt), Gold Coast University Hospital, Southport, Australia.

## Abstract

**Aim:**

To provide an overview of randomized controlled trials (RCTs) in primary total hip arthroplasty summarizing the available high-quality evidence.

**Materials and Methods:**

Following Preferred Reporting Items for Systematic Reviews and Meta-Analyses guidelines (PRISMA), we searched the Cochrane Central Register of Controlled Trials (2020, Issue 1), Ovid MEDLINE, and Embase. We excluded nonrandomized trials, trials on neck of femur fractures or revision surgery, systematic reviews, and meta-analyses. Trials that met our inclusion criteria were assessed using a binary outcome measure of whether they reported statistically significant findings. These were then classified according to the intervention groups (surgical approach, fixation, and component design use, among others).

**Results:**

Three hundred twelve RCTs met the inclusion criteria and were included. The total number of patients in those 312 RCTs was 34,020. Sixty-one RCTs (19.5%) reported significant differences between the intervention and the control groups. The trials were grouped into surgical approach 72, fixation 7, cement 16, femoral stem 46, head sizes 5, cup design 18, polyethylene 25, bearing surfaces 30, metal-on-metal 30, resurfacing 20, navigation 15, robotics 3, surgical technique 12, and closure/drains/postoperative care 13 RCTs.

**Discussion:**

The evidence reviewed indicates that for the vast majority of patients, a standard conventional total hip arthroplasty with a surgical approach familiar to the surgeon using standard well-established components and highly cross-linked polyethylene leads to satisfactory clinical outcomes. This evidence also offers arthroplasty surgeons the flexibility to use the standard and cost-effective techniques and achieve comparable outcomes.

Total hip arthroplasty (THA) is one of the most successful and cost-effective interventions in orthopaedic surgery.^1^ Since the inception of the modern low friction hip arthroplasty by Charnley^2^ at our institute, little has changed in the fundamentals of this operation. However, significant advances have been achieved in metallurgy and manufacturing processes, particularly with the highly cross-linked polyethylene (PE) ensuring excellent long-term outcomes of THA.^3^ Nonetheless, debate continues over the optimal surgical approach, implant fixation, head sizes, or bearing surfaces. National joint registry data play an important role in monitoring implants, measuring performance and survivorship nationwide such as the Scandinavian registries and the United Kingdom national joint registry, which also collects patient-reported outcome measures' data.^4^ However, in clinical research, high-quality randomized controlled trials (RCTs) provide strong evidence for the efficacy of healthcare interventions and inform evidence-based medicine.^5,6^ In particular, RCTs with results demonstrating clinically or statistically significant differences between two interventions indicate a positive effect of one intervention over another.^7,8^ A large number of RCTs have been conducted in THA over the years with only few reporting significant findings reflecting the lack of marginal effects of evaluated surgical interventions.^9^

In this systematic review of the literature, we therefore aim to evaluate published RCTs in primary THAs summarizing the available high-quality evidence.

## Methods

Following Preferred Reporting Items for Systematic Reviews and Meta-Analyses guidelines (PRISMA),^10^ we carried out the electronic searches in January 2018 and updated searches in January 2020. We searched the Cochrane Central Register of Controlled Trials (2020, Issue 1), Ovid MEDLINE (including Epub Ahead of Print, In-Process & Other Non-Indexed Citations, Ovid MEDLINE Daily, Ovid MEDLINE, and Versions) (1946-20 January 2020), and Embase (1980-20 January 2020). We limited our searches to the English language literature. In MEDLINE, we combined the subject-specific search strategy with the sensitivity maximizing version of the Cochrane Highly Sensitive Search Strategy for identifying randomized trials.^11^ The following search strategy was used [(rct OR randomised OR randomized OR “clinical trial” OR blinded OR “controlled trial”).ti,ab*ARTHROPLASTY, REPLACEMENT, HIP/(“total hip replacement*” OR “THA”").ti/Document type Clinical Trial OR Controlled Clinical Trial OR Randomized Controlled Trial].

We examined the titles and abstracts of articles identified in the search as potentially relevant trials. We obtained the full texts of trials that fulfilled our inclusion criteria (i.e., RCTs for THA) and those that were unclear from perusal of the abstracts. We excluded nonrandomized trials, trials on neck of femur fractures/revision surgery, systematic reviews, and meta-analyses. Trials that met our inclusion criteria were assessed by two authors (H.E.M. and S.R.P.) using a binary outcome measure of whether they reported statistically significant findings. These were then classified according to the intervention groups (surgical approach, fixation method, and design) in a narrative review summarizing the evidence. The results were expressed descriptively in numbers and percentages. SPSS 16.0 software (SPSS) was used for descriptive statistical analysis.

## Results

**Figure 1:**
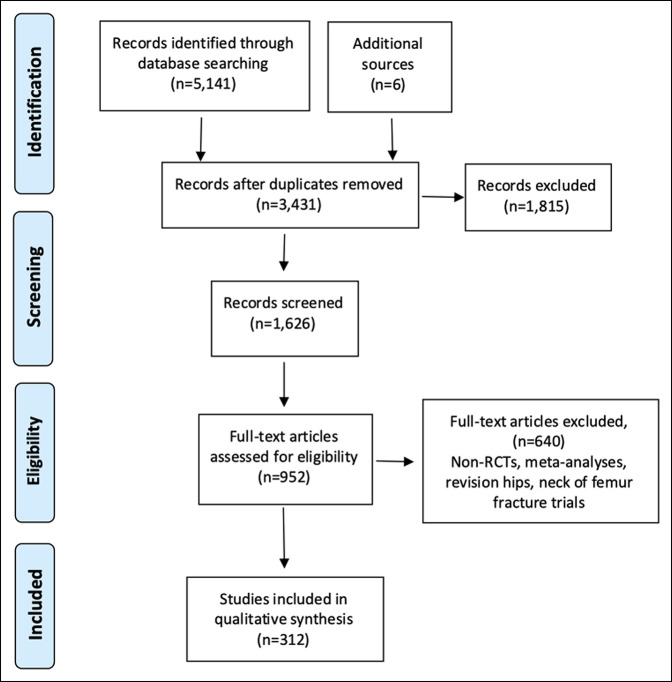
Preferred Reporting Items for Systematic Reviews and Meta-Analyses flow diagram showing electronic searches results and included studies. RCT = randomized controlled trial

**Figure 2:**
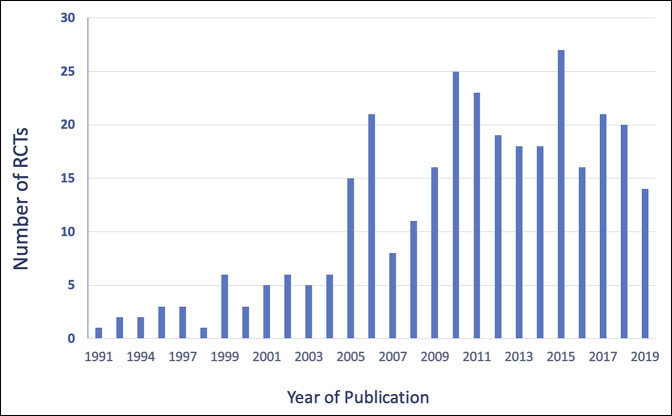
Chart showing the number of RCTs per year of publication. RCT = randomized controlled trial

The electronic searches produced 5141 records, and additional 6 records were identified from reference lists of some included studies. After removing duplicates and screening abstracts, 952 studies were assessed for eligibility, and 312 RCTs met the inclusion criteria and were included (Figure 1). The total number of patients in those 312 RCTs was 34,020. A steady increase has been observed in the number of RCTs published per year with the first conducted in the early 1990s to an average of 20 RCTs per year in the 2010s (Figure 2). Sixty-one RCTs (19.5%) reported significant differences between the intervention and the control groups. The trials were classified according to intervention groups (Table 1).

**Table 1:** The Number of RCTs Classified per Group of Intervention and Percentage of RCTs With Significant Findings

Category	No. of RCTs	No. of RCTs With Significant Findings
Surgical approach	72	5 (6.9%)
Fixation	7	1 (14.3%)
Cement	16	5 (31.3%)
Femoral stem	46	3 (6.5%)
Head sizes	5	1 (20%)
Cup design	18	2 (11.2%)
Polyethylene	25	10 (40%)
Bearing surfaces	30	4 (13.3%)
Metal-on-metal THA	30	20 (66.6%)
Resurfacing	20	1 (5%)
Navigation	15	3 (20%)
Robotics	3	0
Surgical technique	12	5 (41.6%)
Closure, drains, and postoperative care	13	1 (7.7)
Total	312	61 (19.5%)

RCT = randomized controlled trial, THA = total hip arthroplasty

### Surgical Approach

Seventy-two RCTs with 6728 patients evaluated different surgical approaches or related aspects with only five RCTs (6.9%) reporting significant differences between the intervention groups (Table 2). Hamilton et al^12^ evaluated the use of implant positioning software with fluoroscopy in anterior THA in 200 patients and reported closer results to target but with longer operative and fluoroscopy time. Takada et al^15^ compared direct anterior with anterolateral approaches in bilateral THA in 30 patients at a 1-year follow-up focusing on nerve injury and muscle atrophy measured on CT and MRI. They reported no differences in clinical outcomes despite significant differences in muscle atrophy and increased nerve injury with the anterior approach. Acetabular implant positioning was compared radiographically in 60 patients using supine versus lateral patient positioning through a modified Watson-Jones approach with more accurate cup positioning in the supine position.^13^ Moon et al^14^ compared two techniques of posterior soft tissue repair in 167 hips (150 patients) at a 28-month follow-up and reported better outcomes and less dislocations with trans-osseous repair compared with gluteus medius/short rotators tendon-to-tendon repair. Finally, Kruse et al^16^ compared radiographic outcomes of posterior and lateral approaches in 80 patients and reported that the femoral offset and abductor moment arm were significantly increased when using posterior compared with lateral approach.

**Table 2:** Surgical Approaches' Randomized Controlled Trials With Significant Findings

Study	Intervention	Outcome Measures	Results
Hamilton et al^12^	Surgical positioning software with fluoroscopy versus fluoroscopy-alone technique in anterior THA (n = 200)	Cup placement time, total fluoroscopy time, and cup position	Cups placed using software were significantly closer to the target abduction angle (*P* < 0.001) with fewer outliers. Cup placement took longer in the software group (*P* < 0.001), and 2 seconds more total fluoroscopy time (*P* < 0.001).
Takada et al ^13^	Supine versus lateral position using the modified Watson-Jones approach (n = 60)	Cup positioning on radiograph and CT (target abduction 40°)	The supine group was significantly more accurate than lateral group (2.4° versus 4.5°; 95% CI 0.7–3.5; *P* < 0.01). No significant difference in terms of radiographic cup anteversion.
Moon et al ^14^	Transosseous versus gluteus medius tendon (tendon-to-tendon) posterior repair at ∼28.8 months FU (n = 167 hips/150 patients)	Failure of repair using radiopaque markers radiographically, dislocation rate	Transosseous group failure was (18.4%) compared with tendon-to-tendon group (65%; *P* < 0.001). Dislocation rate was significantly higher in the tendon-to-tendon group (7 versus 1.1%; *P* = 0.041).
Takada et al ^15^	Direct anterior (DA) versus anterolateral THA at 1-year FU (n = 30 bilateral)	Lateral femoral cutaneous nerve (LFCN) injury, and tensor fascia lata (TFL) atrophy on CT and MRI	Temporary LFCN injury in DA group only (23.3%). The ratio of the 3-month postoperative to preoperative cross-sectional area of TFL on CT significantly lower on DA side (*P* < 0.01). At 1-year MRI, the mean grade of fatty atrophy of TFL by Goutallier classification was significantly higher in DA (*P* = 0.03). No significant difference in clinical outcomes between both sides at postoperative 1 year.
Kruse et al ^16^	Posterior versus lateral approach (n = 80)	Radiographic cup position, femoral offset, abductor moment arm, and leg length discrepancy between the two approaches	Mean anteversion was 5° larger in the posterior approach (95% CI, −8.1 to −1.4; *P* = 0.006). Mean inclination was 5° less steep (95% CI, 2.7–7.2; *P* < 0.001) compared with the lateral approach. The posterior approach had a larger mean femoral offset of 4.3 mm (95% CI, −7.4 to −1.3, *P* = 0.006), mean total offset of 6.3 mm (95% CI, −9.6 to −3; *P* < 0.001), and mean abductor moment arm of 4.8 mm (95% CI, −7.6 to −1.9; *P* = 0.001) compared with the lateral approach. Femoral offset and abductor moment arm were significantly increased when using the posterior approach.

CI = confidence interval, THA = total hip arthroplasty

Twenty-two RCTs looked specifically at minimally invasive surgery techniques and compared the outcomes with standard techniques including anterior, anterolateral, and posterior approaches; none have reported significant differences in their measured outcomes. The remaining 45 RCTs were as follows (Appendix 1): mini-incisions and two-incision approaches in 13 RCTs, anterior versus posterior approaches in 9 RCTs, anterior versus lateral approaches in 7 RCTs, lateral versus posterior approaches 4 RCTs, variant posterior approaches such as repair of soft tissues or not in 4 RCTs, piriformis sparing approach in 2 RCTs, use diathermy and electrocautery in 2 RCTs, and one RCT for each supercapsular percutaneously assisted approach, trans-trochanteric approach, outpatient anterior approach and patient positioning; none of these 45 RCTs reported any significant differences.

### Fixation (Cemented Versus Cementless) of Total Hip Arthroplasty

Seven RCTs compared cemented and cementless THA of different brands with a total of 1271 patients. Only one trial, Corten et al,^17,18^ reported significantly better survivorship for cementless THA in their 20-year follow-up report of their 93 patients from an original RCT sample size of 250 patients (*P* = 0.020). The cementless tapered stem had an extremely good survival rate of 99%. Radiographs showed evidence of mild stress-shielding around 95% of the cemented stems and 88% of the cementless stems; stress-shielding of grade 3 or greater was seen around the remaining 12% of the cementless stems. The remaining six RCTs reported no significant differences between cemented and cementless THA although their follow-up was only up to 5 years.

### Cement Trials

Sixteen RCTs with a total of 979 patients evaluated cement comparing different viscosities or different types of cement restrictors with five RCTs (31.3%) reporting significant findings (Table 3). Koessler et al^19^ evaluated a modified cementing technique to reduce the intramedullary pressure in 120 patients and measured the embolic events using continuous perioperative transesophageal echocardiography reporting significant embolic events with conventional cementing techniques although no patient developed frank fat embolism syndrome. Visser et al^20^ compared three types of cement restrictors in 93 patients measuring their postoperative radiographs efficiently and reported significant failures with the Biosem restrictor (SEM). Degradable cement restrictors were reported to have significantly worse outcomes in three RCTs. Freund et al^21^ reported more failures with a resorbable restrictor with a longer cement plug although no difference was observed in stem loosening at a 2-year follow-up. Schauss et al^22^ also reported a shorter cement plug with nondegradable plugs in 130 patients compared with degradable restrictors. Finally, Wembridge et al^23^ compared PE with biodegradable cement restrictors in 32 patients and reported worse migration and longer cement plug with the biodegradable restrictors.

**Table 3:** Cement Randomized Controlled Trials With Significant Findings

Study	Intervention	Outcome Measures	Results
Koessler et al^19^	Conventional cemented versus modified cemented THA (vacuum drainage placed in the proximal femur to reduce the increase of intramedullary pressure during insertion of the prosthesis) (n = 120)	Embolic events detected by continuous transesophageal echocardiography (TEE) hemodynamic monitoring and blood gas analysis were done during the perioperative period.	Significantly more embolic events with the conventionally cemented group (93.3% versus 13.3% *P* < 0.05). No clinical signs of fat embolism syndrome in any study patient.
Visser et al^20^	Biosem, Cemlock, or Thackray cement plugs in Stanmore hip prosthesis THA (n = 93)	Occlusion and stability on postop radiographs	Significantly more failures with Biosem: The percentages of deficient plugs were Biosem 78% (25/32), Cemlock 32% (9/28), and Thackray 18% (6/33). Comparison of the smaller sizes of the prosthesis versus the larger sizes showed a significant effect on the stability of the plugs.
Freund et al^21^	Resorbable (Shuttle stop) versus nonresorbable polyethylene cement restrictor at 2-year FU (n = 70)	Migration of the restrictor, cement leakage, and possible early aseptic loosening	More failures with displacement or leakage of the resorbable restrictor (3 versus 16; *P* < 0.01). No differences in stem loosening or grade of radiolucent lines at 2 years.
Schauss et al^22^	Degradable versus nondegradable cement restrictor (n = 130)	Distal migration during stem insertion, radiographs	Better stability with nondegradable plugs Cement plug length 27 versus 15 mm (*P* = 0.003).
Wembridge et al^23^	UHMWPE versus biodegradable cement restrictor (n = 32)	Postoperative radiographs restrictor migration	Worse results with biodegradable restrictor: Mean migration was 3.0 versus 0.5 cm (biodegradable versus UHMWPE, *P* < 0.002).

UHMWPE = ultra-high-molecular-weight polyethylene

The remaining 11 RCTs reported no significant differences, including one RCT comparing *Hardinge* cement restrictor with an autogenous bone plug restrictor, one RCT compared the thickness of the cement mantle (thin versus thick), three RCTs comparing fluoride-containing acrylic cement with conventional cement, Palacos, or Palacos G cement. Additional six RCTs made the following comparisons between different types of cement with no significant differences reported; low/medium Simplex P cement versus high-viscosity Simplex AF cement (*Stryker-Howmedica)*, Cemex Rx *(Cemex System, Tecres, S.p.A.)* versus Palaces R cement *(Schering Plough, Labo NV)*, Palamed G *(Biomet Merck)* versus Palacos R cement *(Schering Plough, Labo NV),* SmartSet HV *(*DePuy *CMW)* versus Palacos R cement *(Schering Plough)*, Palacos R (*Schering Plough)* versus Palacos R + G *(Schering-Plough)*, and Palacos versus Palamed cement *(Biomet Merck)*.

### Femoral Stems Trials

Forty-six RCTs with 5242 patients evaluated aspects specifically related to femoral stems. Only three RCTs reported significant differences. Berger et al^24^ reported a significantly lower rate of cement mantle deficiencies when using stem centralizer in 60 patients (*P* < 0.001). In their trial of 39 patients at a 2-year follow-up, Tanzer et al^25^ assessed femoral bone remodeling using dual-energy radiograph absorptiometry after a titanium proximally porous-coated femoral implant with or without hydroxyapatite (HA)-tricalcium phosphate coating. The HA-tricalcium phosphate-coated stems had significantly less femoral bone loss. Luites et al^26^ compared 22 titanium and 20 HA-coated ProxiLock stems at a 2-year follow-up and reported significantly higher early failures with the ProxiLock stems requiring revision surgery.

The remaining 43 trials reported no significant differences (Appendix 1). These included eight RCTs comparing different cemented stems, nine RCTs comparing uncemented stems, three RCTs comparing collared versus collarless stems, six RCTs evaluated HA-coated stems, four RCTs evaluated porous-coated stems, nine RCTs evaluated short stems, and three RCTs looked at different preparation techniques of femoral stems.

### Head Sizes

Five RCTs compared different head sizes in 889 patients. Only one large multicentre RCT, Howie et al,^27^ reported significant findings. They compared the dislocation rate between 28 and 36 mm metal heads on highly cross-linked polyethylene (HXLPE) at a 1-year follow-up in 533 patients with primary THA: 4.4% (12/275) versus 0.8% (2/258) (95% confidence interval, 0.9% to 6.8%) (*P* = 0.024). The remaining four RCTs reported no significant differences including 28 versus 32 mm ceramic-on-ceramic (CoC) THA; 32 versus 36 mm ceramic-on-PE THA, 28 versus 36 mm metal-on-cross-linked PE, and small head 28 mm metal-on-metal versus metal-on-PE.

### Cup Design

Eighteen RCTs with 1778 patients evaluated aspects of acetabular component designs with two RCTs reported significant findings (11.2%). Faris et al^28^ evaluated the use of all-PE cemented cups (407 THA) with or without integrated cement spacers attached at the back of the cup to ensure a uniform cement mantle. They reported a significantly higher rate of failure with integrated spacers compared with no spacers at a 6.5-year follow-up. Stilling et al^29^ compared titanium uncemented cups with first-generation HA-coated cups at a 15-year follow-up with a significantly higher revision rate for HA-coated cups.

The remaining 16 RCTs were a heterogeneous group that made the following comparisons with no significant differences between the interventions in either clinical or radiographic outcomes, including solid versus cluster hole cups; scientific versus Omnifit cups; tantalum versus titanium cups; porous tantalum monoblock cup versus porous-coated titanium monoblock cup; trabecular metal cups versus titanium fiber-mesh cups; porous titanium versus conventional titanium cups; solid-backed versus cluster-hole cups all without screws; HA-coated versus porous-coated cups; cementless cup with or without screws; uncemented 61% high porosity versus 45% low porosity cups; BICON-PLUS versus BICON-PLUS NT cup; finger-packing versus cement pressurization cemented cups; cemented Charnley versus uncemented Duraloc 1200 cups; cemented PE versus uncemented porous-coated cups; cemented cups versus porous-coated cups, and all-poly press-fit RM cup with or without screw fixation.

### Polyethylene Trials

Twenty-five RCTs with 2216 patients compared different types of PE particularly the effect of cross-linking on wear rates with a long-term follow-up (multiple publications). Ten RCTs (13 studies; Table 4) reported significant differences. Cross-linked polyethylene showed better wear characteristics compared with conventional PE at 5- and 10- and 15-year follow-ups.^30–32^ Similarly, HXLPE consistently shown to have significantly better wear characteristics across different trials up to a 12-year follow-up^33,34,35,36,37,38,39,40^ (Table 4). Vitamin E-infused HXLPE was also shown to have significantly better wear rates across two RCTs with a 3-year follow-up compared with ultra-high-molecular-weight polyethylene (UHMWPE).^41,42^ The remaining 12 studies reported no significant differences and made the following comparisons: cross-linked polyethylene versus PE in three trials; HXLPE versus PE in four trials; Vitamin E-infused HXLPE versus HXLPE in four trials; and one trial compared Sulene-poly *(Sulene; Zimmer GmBH)* versus Durasul-poly liner *(Durasul; Zimmer that was sterilized by ethylene oxide)* with no significant differences (Appendix 1).

**Table 4:** Polyethylene Randomized Controlled Trials With Significant Findings

Study	Intervention	Outcome Measures	Results
Geerdink et al^30^	XLPE versus conventional PE at 5-year FU (n = 127/133 hips)	Polyethylene wear rates	Better results with cross-linked at a mean wear rate of 0.083 (SD 0.056) versus 0.123 (SD 0.082) mm/yr.
Engh et al^31^	XLPE versus conventional PE at 10-year FU (n = 185)	Revision for wear-related complications.	Better survivorship at 10 years for XLPE 100% versus 94.7% (*P* = 0.003). For unrevised hips, the mean linear wear rate was 0.22 versus 0.04 mm/yr for XLPE (*P* < 0.001).
Hopper et al^32^	XLPE versus conventional PE THA at 15 years (n = 85 hips) (230 hips/220 patients at the beginning of the trial)	THA wear, osteolysis, revision rate, radiographic follow-up	Cumulative incidence of revision at 15 years using reoperation for wear-related complications as an end point was lower in the XLPE group (0% versus 12%; *P* < 0.001). Among unrevised THAs with a minimum 14-year radiographic follow-up: The mean steady-state linear wear rate for XLPE (0.03 ± 0.05 versus 0.17 ± 0.09 mm/yr *P* < 0.001).
Martell et al^33^	HXLPE versus conventional polyethylene (PE) at 2- to 3-year FU (n = 46)	Polyethylene wear rates	A significant reduction in 2- and 3-dimensional linear wear rates (42% and 50%) was found with the HXLPE group (*P* = 0.001 and *P* = 0.005).
Glyn-Jones et al^34^	HXLPE versus conventional PE at 3-year FU (n = 54)	RSA analysis, creep and wear behavior	Less wear with HXLPE with mean total penetration 0.35 mm (SD 0.14) for HXLPE versus 0.45 mm (SD 0.19) (*P* = 0.0184). Significant difference (*P* = 0.012) in the mean wear rate for HXLPE was 0.03 (SD 0.06) versus 0.07 (SD 0.05) mm/yr.
Thomas et al^35^	HXLPE versus conventional PE at 7-year FU (n = 54)	Wear rate, RSA	Mean total femoral head penetration was significantly lower in HXLPE 0.33 versus 0.55 mm (*P* = 0.005). The mean steady-state wear rate of HXLPE was 0.005 versus 0.037 mm/yr (*P* = 0.007).
Glyn-Jones et al^36^	HXLPE versus conventional PE at 10-year FU (n = 39/54)	RSA wear, OHS	Significantly less wear rate with the HXLPE group 0.003 (SD 0.023) versus 0.030 (SD 0.0.27) mm/yr. Volumetric penetration from 1 to 10 years for the UHMWPE group was 98 versus 14 mm (*P* = 0.01).
Broomfield et al^37^	HXLPE versus conventional PE at 12-year FU (n = 25/54)	Periacetabular osteolysis, CT	Significantly lower incidence of periacetabular osteolysis in the HXLPE group (*P* = 0.042)
Calvert et al^38^	HXLPE versus conventional PE at 4-year FU (n = 119)	Linear 3D and volumetric wear	Linear, 3-dimensional, and volumetric wear rates were significantly less in HXLPE (*P* < 0.05).
Mutimer et al^39^	HXLPE versus conventional PE at 5-year FU (n = 122)	Radiographs, wear rate	The 2D wear rate for HXLPE was significantly less than standard poly 0.05 versus 0.26 mm/yr (*P* < 0.001).
Langlois et al^40^	HXLPE versus moderately XLPE in cemented component at 8-year FU (n = 68)	Clinical outcomes, wear rates	Better wear rates with HXLPE: The rate of penetration from one year onward was 0.0002 versus 0.1382 mm/year (*P* < 0.001).
Scemama et al^41^	HXLPE/Vitamin E-infused versus UHMWPE hybrid THA at 3-year FU (n = 74)	Femoral head penetration radiographically	Better wear rates with the Vitamin E group Median creep 0.111 versus 0.170 mm (*P* = 0.046). Median steady-state penetration rate 0.008 versus 0.133 mm/year (*P* = 0.043).
Rochcongar et al^42^	HXLPE/Vitamin E-infused versus UHMWPE cups at 3-year FU (n = 62)	RSA wear rate	The cumulative penetration after 3 years was 0.200 mm for the HXLPE/Vitamin E cup versus 0.317 mm for the UHMWPE cup (*P* < 0.0001).

PE = polyethylene; THA = total hip arthroplasty, HXLPE = highly cross-linked polyethylene, XLPE = cross-linked polyethylene, UHMWPE = ultra-high-molecular-weight polyethylene, RSA = radiostereometric analysis, OHS = Oxford Hip Score

### Bearing Surfaces

Thirty RCTs with 5425 patients compared different bearing surfaces in THA with only four RCTs (13.3%) reporting significant findings (Table 5). Kim^43^ compared the PE wear rate between zirconia head and cobalt chromium heads in sequential bilateral THAs in 52 patients at a 7.1-year follow-up and reported lower wear rates with zirconia heads. von Schewelov et al^44^ compared four different articulations of 22.225 mm heads made from zirconium oxide ceramic or stainless steel, articulating against either standard UHMWPE or *Hylamer*; a modified-UMWPE, in 114 patients at a 5-year follow-up. They reported worse outcomes with zirconium oxide heads/*Hylamer* and advised against their use. *Hylamer* was later withdrawn from the market due to the high failure rate. Vendittoli et al,^45^ in a long-term RCT, compared conventional metal-on-PE articulations with alumina on alumina ceramic bearings with significantly better outcomes in favor of ceramic bearings. Finally, Atrey *et al*,^46^ in their 10-year follow-up trial of different bearing surfaces including ceramic-on-ceramic (CoC), reported a less wear rate with metal-on-cross-linked polyethylene compared with metal-on-UHMWPE.

**Table 5:** Bearing Surfaces' Randomized Controlled Trials With Significant Findings

Study	Intervention	Outcome Measures	Results
Kim ^43^	Zirconia head versus cobalt-chromium head in bilateral THA at 7.1-year FU (n = 52)	Polyethylene wear, radiographic evaluations	Significantly lower wear with zirconia heads: The mean polyethylene wear rate was 0.08 mm/yr with zirconia heads versus 0.17 mm/yr with cobalt-chromium heads (*P* = 0.004). Volumetric wear 350.8 versus 744.7 mm^3^ (*P* = 0.004).
von Schewelov et al^44^	4 articulations: Stainless steel/Enduron, stainless steel/Hylamer cup, zirconium oxide ceramic/Enduron, or zirconium oxide ceramic/Hylamer at 5-year FU (n = 114)	Wear and migration RSA analysis	Mean annual wear 0.11 mm for a stainless steel/Enduron articulation, 0.34 mm for stainless steel/Hylamer cup, 0.17 mm for zirconium oxide ceramic/Enduron, and 0.40 mm for zirconium oxide ceramic/Hylamer. The difference between the groups was significant (*P* < 0.008) except for stainless steel/Hylamer versus zirconium oxide ceramic/Hylamer (*P* = 0.26). Zirconium oxide ceramic femoral head should not be used with a polymethylmethacrylate acetabular component.
Vendittoli et al^45^	Metal-on-poly versus alumina on alumina bearings at 9- to 15-year FU (n = 107 hips)	Reoperation, revision rate, radiological outcomes (UCLA, WOMAC)	Better outcomes with ceramic bearings: Revision rate for aseptic loosening or wear 11.6% versus 1.4% (*P* = 0.017). Significant difference in the UCLA score in favor of ceramic bearings (5.6 versus 4.8, *P* = 0.015). No significant difference in for WOMAC score.
Atrey et al^46^	UHMWPE/metal head, XLPE/metal head, or ceramic-on-ceramic at 10-year FU (n = 97 hips)	Radiological analysis of wear, HHS, WOMAC, SF-12	Significantly reduced rate of linear wear with XLPE (0.07 mm/yr) compared with UHMWPE (0.37 mm/yr) (*P* = 0.001). Volumetric wear was also significantly reduced in the XLPE group (29.29 mm^3^/yr) compared with the UHMWPE group (100.75 mm^3^/yr) (*P* = 0.0001). THHS was significantly less in the UHMWPE group (*P* = 0.0188) than in the other two groups. No difference in WOMAC or SF-12 between the groups.

THA = total hip arthroplasty, XLPE = cross-linked polyethylene, UHMWPE = ultra-high-molecular-weight polyethylene, HHS = Harris Hip Score

The remaining 26 RCTs reported no significant differences including 10 RCTs comparing CoC with ceramic-on-polyethylene bearings, 11 RCTs comparing CoC with metal-on-polyethylene bearings, four RCTs comparing metal-on-polyethylene with ceramic-on-polyethylene bearings and one trial compared different polyethylene liners with metal heads.

### Metal-on-Metal Total Hip Arthroplasty

Thirty RCTs compared metal-on-metal (MoM) THA with other bearing surfaces in 2912 patients. This was a unique group of trials where nearly all RCTs that looked at metal ions in their reported outcomes found statistically significant higher levels of ions with MoM but similar clinical outcomes and patient-reported outcome measures. Trials that did not report on metal ion levels (10 RCTs) found no significant differences in their reported outcomes comparing MoM with other bearings (Appendix 1).

### Hip Resurfacing Versus Total Hip Arthroplasty

Twenty RCTs looked at hip resurfacing in 1762 patients. Only one RCT (5%) reported statistically significant differences. Penny et al^47^ compared Articular Surface Replacement (ASR) hip resurfacing prosthesis with THA at a 2-year follow-up in 38 patients and found higher consistently higher metal ions levels with ASR (*P* ≤ 0.001). The remaining 19 trials reported no statistically significant differences although the majority were short-term follow-ups (2 to 5 years). These included 15 RCTs comparing outcomes of hip resurfacing versus THA; two RCTs compared hip resurfacing with MoM THA; one RCT compared cemented versus cementless femoral stem; and one RCT compared posterior versus anterolateral approach in hip resurfacing (Appendix 1).

### Navigation and Robotics

Navigation was evaluated in 15 RCTs with a total of 1158 patients. Three RCTs (20%) reported significant differences with improved cup positioning (Table 6). The remaining 12 RCTs reported no significant differences including navigated versus free hand techniques for THA in 10 RCTs, one RCT compared fluoroscopy versus imageless navigation minimally invasive techniques, and one RCT compared navigated versus standard hip resurfacing (Appendix 1).

**Table 6:** Navigation Randomized Controlled Trials With Significant Findings

Study	Intervention	Outcome Measures	Results
Kalteis et al^70^	Free-hand versus computer assistance image-free navigation cup positioning (n = 45)	CT scans for cup position	More accurate positioning with navigation and deviations from the desired cup position (45° inclination, 15° anteversion) were significantly lower in the computer-assisted study group (*P* < 0.001).
Verdier et al^71^	NAVEOS navigation versus freehand cup placement THA at 3-month FU (n = 78)	CT cup position measurements (safe zone: 15° ± 10° radiological anteversion and 40° ± 10° radiological inclination)	Better cup positioning with navigation: Cups in the safe zone were 67% versus 38% (*P* = 0.012). Navigation was discontinued prematurely in 6 patients (intention-to-treat analysis used). Complications: 1 dislocation and 1 infection, both in the freehand group.
Yamada et al^72^	CT-based 2D-3D navigation versus paired-point matched navigation group (PPM) (n = 80)	Accuracy of cup orientation (absolute difference between the intraoperative record and the postoperative measurement)	Better accuracy with CT-based 2D-3D matched navigation: Accuracy of cup inclination 2.5° ± 2.2° versus 4.6° ± 3.3° (*P* = 0.0016). Accuracy of cup anteversion 2.3° ± 1.7° versus 4.4° ± 3.3° (*P* = 0.0009)

THA = total hip arthroplasty.

Furthermore, three RCTs evaluated the use of robotics in THA in 275 patients. In their early robotic RCT, in 2003, Honl et al^48^ randomized 154 patients to conventional or robotic-assisted THA and compared 2-year outcomes using Harris, Merle d'Aubigné, and the Mayo scores with no significant differences reported. However, the duration of robotic procedures was longer with 18% of attempted robotic implantations converted to manual implantations as a result of system failure. Dislocation was more frequent with robotics 11/61 versus 3/80 (*P* < 0.001) as well as revision surgery 8/61 (*P* < 0.001). Lim et al^49^ evaluated the effects of robotic milling versus manual rasping on the accuracy of short femoral stem positioning and on the clinical outcomes in 54 patients at a 2-year follow-up and reported no significant differences. Finally, Bargar et al^50^ reported a mean 14-year follow-up outcomes of 67 patients from 2 U.S. Food and Drug Administration trials who underwent conventional versus active robotic system THA. No statistically significant difference was observed in probability of a revision for wear or loosening. The robotic group had statistically significant higher Health Status Questionnaire pain and Harris pain scores but lower Western Ontario and McMaster Universities Osteoarthritis Index (WOMAC) scores.

### Surgical Technique and Miscellaneous Trials

Twelve heterogeneous RCTs looking at surgical technical aspects of THA are presented in this group with 1098 patients. Five RCTs (41.6%) reported significant findings in favor of using a measuring device to minimize leg length discrepancy,^51^ high-efficiency particulate air to reduce colony-forming units within 5 cm of the surgical wound,^52^ better acetabular component positioning measured on postoperative CT scan with the use of patient-specific instrumentations,^53^ the use of transverse acetabular ligament for cup anteversion and inclination,^54^ and the use of digital inclinometer-assisted cup insertion technique^55^ (Table 7).

**Table 7:** Surgical Techniques Randomized Controlled Trials With Significant Findings

Study	Intervention	Outcome Measures	Results
Bose et al^51^	THA with or without measuring device for leg length discrepancy (n = 117)	Leg length discrepancy radiographs	Statistically significant decrease in limb-length inequality with the use of measuring device average LLD 8.8 versus 3.4 mm (*P* < 0.01).
Stocks et al^52^	Directed air flow high-efficiency particulate air (HEPA), system present but switched off or control filter during THA (n = 36)	Airborne particulate, colony-forming units within 5 cm of surgical wound	All particulate and bacterial counts at the surgical site were significantly lower in the directed air flow group (*P* < 0.001).
Small et al^53^	Patient specific versus standard surgical instruments THA (n = 36)	Acetabular shell position on CT scan	Better implant positioning with intervention group; differences found between planned and actual anteversion were −0.2° ± 6.9° for PSI versus −6.9° ± 8.9° (*P* = 0.018).
Meermans et al^54^	Freehand versus transverse acetabular ligament reference for acetabular anteversion (n = 80)	Radiographic measurement of anteversion and inclination.	Better component positioning using TAL as a reference: Anteversion: 21° (2° to 35°) versus 17° (2° to 25°) (*P* = 0.004). Inclination: No significant difference between the two techniques although less outliers (safe zone) with TAL.
O'Neill et al^55^	Freehand, modified Mechanical Alignment Guide (MAG) or digital inclinometer-assisted cup insertion techniques (n = 270)	Postoperative radiographic cup inclination as measured by target to apparent operative inclination (AOI 35° ± 2.5°)	Digital inclinometer technique achieved AOI target in 88% versus 71% of MAG versus 51% Freehand. Statistically significant differences between: Freehand versus inclinometer groups (*P* < 0.001) Freehand versus MAG (*P* < 0.001) Digital inclinometer versus MAG (*P* < 0.023).

THA = total hip arthroplasty, LLD = leg length discrepancy

The remaining seven RCTs reported no significant differences and made the following comparisons: sequential versus simultaneous bilateral THA; removal versus retention of subchondral bone plate for cemented cups in two trials; cup insertion with or without inclinometer; the use of abductor shuck versus trans-osseous pins (a level-caliper system using trans-osseous periacetabular and femoral pins as two fixed points) versus patella electrocardiogram leads to measure intraoperative leg length; plasma-rich platelets versus no plasma-rich platelet in bilateral THA; and autologous impaction bone grafting versus traditional technique in cementless THA (Appendix 1).

### Skin Closure, Drain, and Postoperative Care

There were 13 RCTs in this group with 2287 patients included. Only one RCT (7.7%) reported significant findings. Rui et al^56^ compared staples versus absorbable subcuticular suture for skin closure at a 3-month follow-up in 165 patients. They reported no infections in sutures group versus 2 superficial infections (2.4%) in the staples group. A statistically significant difference was observed in favor of the suture group for time to dry surgical incisions (4.8 versus 5.0 days, *P* = 0.028), hospital stay (6 versus 12, *P* < 0.001), and cost saving $82.2 per case. Although shorter surgical time to use staples (24.7 versus 357.7 seconds, *P* < 0.001), no difference was observed in patients' satisfaction. However, two additional RCTs made similar comparisons and reported no significant difference between staples and sutures (Appendix 1).

Four RCTs evaluated the use of surgical drain postoperatively comparing it to no drain and reported no significant differences in their measured outcomes. Different postoperative care instructions were also evaluated in six RCTs with no significant differences including weight-bearing status after cementless THA (unrestricted versus protected) across four RCTs and hip precautions after the posterolateral approach in two RCTs (Appendix 1).

## Discussion

In this study, we provide a comprehensive overview of 312 RCTs in primary THA. The total number of patients included in those RCTs was 34,020. The most important finding is that only 19.5% of trials reported significant differences between the intervention and the control groups for the outcome measures used by those trials.

Different surgical approaches were evaluated in 72 trials, the largest subgroup of trials, with ∼93% reporting no significant differences in their reported outcomes. This evidence supports surgeons' preference based on their familiarity with a particular approach that allows adequate exposure to perform THA safely acknowledging that each surgical approach has its own pros and cons. The majority of modern THA cementless acetabular components are hemispheric press-fit with improved modular liner congruity and fixation. Furthermore, the use of HXLPE liners seems to have substantially reduced wear rate and osteolysis. This was a consistent finding in a large number of RCTs included. Fixation of THA and stem designs, once fiercely debated topics, are covered by a variety of RCTs with no clear advantage of the comparators. Forty-six RCTs evaluated various designs of femoral stems, both cemented and cementless, with similar clinical outcomes reported at short to medium term. Here lies one of the limitations of RCT evidence where long-term survivorship data, most pertinent to stem survivorship, are lacking.

In total, 60 RCTs compared different bearing surfaces including metal-on-metal bearings that have consistently shown raised levels of metal ions and the familiar mode of failure of this particular bearing. The evidence reviewed equally supports the use of metal-on-PE, CoC, and ceramic-on-PE bearings; the latter is further supported by emerging long-term survivorship and registry data.^3,57^ Clinical outcomes of hip resurfacing were evaluated in 20 trials in comparison with THA, and functional outcomes were similar at short- to medium-term follow-ups. Trials of navigation techniques show no difference in clinical outcomes although some reported significant differences in radiological outcomes, particularly cup positioning, and a long-term follow-up is needed to see whether this leads to improved clinical outcomes. Finally, skin closure techniques, use of drains, and postoperative weight-bearing status or hip precautions were evaluated in a small number of trials with no significant differences.

Evidence derived from RCTs is based on highly selective populations in a tightly controlled settings and deemed to have the highest reliability. However, most RCTs are short or medium term as obtaining a long-term follow-up is complicated by cost, co-intervention, loss to follow up, and postrandomization variables.^58^ Long-term observational studies and data registries, despite their inherent limitations, prove more practical in evaluating long-term outcomes of THA such as survivorship and reoperations and provide a pragmatic overview of clinical practice.^59–61^ In its 16th annual report, the UK's national joint registry has collated data for over 1 million primary THA with up to a 15-year follow-up. Ceramic-on-polyethylene bearings performing particularly well and the overall revision rates after primary THA have reduced over the last 10 years after the peak of metal-on-metal bearings.^57^ Similar trends have been reported in other national registries and long-term follow-up studies^3^; the RCTs included in this study support those findings.

Patient-reported outcome measures (PROMs) play an important role in evaluating interventions in terms of outcomes that matter to patients and widely used in clinical research.^62^ The majority of trials included in this study used PROMs (Oxford Hip Score, Harris Hip Score, WOMAC) as a primary or secondary measure. A number of studies have demonstrated a ceiling effect of those PROMS where a considerable proportion of patients score the best/maximum or worst/minimum score, making the measure unable to discriminate between subjects at either extreme of the scale.^63,64^ However, more recent registry-based observational studies have demonstrated that population-wide data do not exhibit a ceiling or floor effect of these PROMs.^65^ Others have found only weak-to-moderate correlation between PROMs and patient satisfaction.^66^ The International Society of Arthroplasty Registries PROMs working group acknowledges the variation in the specific PROMs used and does not make specific recommendations about which PROMs to use in arthroplasty registries.^67^ PROMs are used in many registries to support quality assurance and provide information on value-based care. However, in the context of RCTs, they may not detect the marginal effects of the evaluated interventions.

This is the first study to undertake a comprehensive overview of RCTs in THA. We do, however, acknowledge limitations to its findings. We did not calculate the treatment effect of individual trials with significant statistical findings and whether this correlated with clinically measurable effects. Furthermore, the quality of reporting trials was not addressed as this aspect falls outside the scope of this study. However, reporting bias or publication bias in clinical research is a known phenomenon where data from trials with negative findings are not publicized, and so they remain inaccessible.^68^ The prospective registration of trials and public access to study data via results databases had been introduced to minimize the impact reporting bias.^69^ The true scale of this bias in the clinical literature is unclear. However, ∼80% of published RCTs in THA reported no significant differences “negative trials,” which may indicate that there is no tendency to overestimate the efficacy and underestimate the risks of the interventions evaluated in those trials.

To conclude, THA is a successful and durable operation that has helped millions of patients worldwide. The early failures encountered in the 1970 to 1980s had been largely addressed in the 1990s and the early 2000s with improved metallurgy and manufacturing processes. The RCT evidence presented indicates that for the vast majority of patients, a standard conventional THA with a surgical approach familiar to the surgeon using standard well-established components and highly cross-linked polyethylene leads to satisfactory clinical outcomes. This evidence also offers arthroplasty surgeons the flexibility to use the standard and cost-effective techniques and achieve comparable outcomes. Future trials should also focus on preoperative interventions to improve clinical outcomes, an area that is currently lacking in THA trials.

## Appendix 1 Trials With No Significant Findings

**Table:**

Category	Studies
Surgical approach	Brismar 2018, Nistor 2017, De Anta-Díaz 2016, Restrepo 2010, Zomar 2018, Mjaaland 2015, Brun 2019, Taunton 2018, Barrett 2019, Bon 2019, Rykov 2017, Zhao 2017, Cheng 2017, Christensen 2015, Taunton 2014, Barrett 2013, Widman 1999, Morris 2013, Rosenlund 2017, Rosenlund 2016, Ji 2012, Witzleb 2009, Stevenson 2017, Shitama 2009, Speranza 2007, Ogonda 2005, Dienstknecht 2014, Reichert 2018, Tan 2019, Krych 2010, Pagnano 2009, Pagnano 2008, Abdel 2017, Sershon 2017, Hu 2012, Goyal 2017, Khan 2012a, Khan 2012b, Pace 2008, Ouyang 2018, Horwitz 1993, Tarasevicius 2011, Tarasevicius 2010, Tarasevicius 2006, Chiu 2000, Chimento 200, Lawlor 2005, Kim 2006, Meneghini 2008, Wohlrab 2008, Mazoochian 2009, Meneghini 2009, Della Valle 2010, Müller 2010, Pospischill 2010, Varela-Egocheaga 2010, Yang 2010, Foucher 2011, Goosen 2011, Martin 2011, Müller 2011, Dienstknecht 2013, Greidanus 2013, Varela-Egocheaga 2013, Petridis 2014, Biau 2015, Repantis 2015
Fixation	Wykman 1991, Laupacis 1993, Rorabeck 1996, Kim 2002, Mulliken 1996, Grant 2005
Cement	Jeffery 1997, Nivbrant 2001, Digas 2004, Digas 2005, Nelissen 2005, Digas 2006, Hallan 2006, Husby 2010, Van Der Voort 2016, Meinardi 2016, van IJperen 2018
Stem	Rasquinha 2004, Lachiewicz 2008, McCalden 2010, Hutt 2014, Marston 1996, Nivbrant 1999, Thien 2010, Kadar 2011, Ström 2006, Johnston 2001, Karachalios 2004, Healy 2009, Simpson 2010, Nysted 2011, Bennett 2014, Miyatake 2015, Van Oldenrijk 2017, Meding 1997, Meding 1999, Settecerri 2002, Ciccotti 1994, Kärrholm 1994, Incavo 1998, Yee 1999, Yoon 2007, Camazzola 2009, Kärrholm 2002, MacDonald 2010, Baad-Hansen 2011, Sandiford 2014, Gielis 2019, von Roth 2014, Salemyr 2015, Freitag 2016, Kim 2016, Koyano 2017, Schilcher 2017, Ferguson 2018, Samy 2019, Laupacis 2002, Hjorth 2016, Pitto 1999
Head sizes	Lee 2014, Lindalen 2015, Howie 2016, van der Veen 2019
Cup design	Flivik 2005, Digas 2006, Bjørgul 2010, Baad-Hansen 2011, Angadi 2012, Pakvis 2012, Ullmark 2012, Veldstra 2012, Naudie 2013, Broeke 2013, Ayers 2015, Blakeney 2015, Salemyr 2015, Wegrzyn 2015, Minten 2016, Gallen 2018.
Polyethylene	Engh 2006, García-Rey 2008, Ayers 2009, Geerdink 2009, McCalden 2009, Jonsson 2015, Salemyr 2015, Nebergall 2017, Shareghi 2015, Devane 2017, Teeter 2017, Galea 2019
Bearing surfaces	Amanatullah 2011, Atrey 2018, Bascarevic 2010, Beaupre 2013, Beaupre 2016, Borgwardt 2017, Cai 2012, Capello 2005, Capello 2005, Capello 2008, D'Antonio 2002, D'Antonio 2005, Hamilton 2010, Ise 2009, Jassim 2015, Kadar 2011, Kim 2013, Lewis 2010, Lombardi 2010, Morison 2014, Nikolaou 2012, Pitto 2008, Sonny 2005, Vendittoli 2007, Zerahn 2011, Zhou 2006
Metal-on-metal THA	Lombardi 2001, Brodner 2003, MacDonald 2003, Jacobs 2004, Lombardi 2004, Nygaard 2004, Grübl 2006, Vendittoli 2006, Dahlstrand 2009, Engh 2009, Garbuz 2010, Hailer 2011, Malviya 2011, Weissinger 2011, Zijlstra 2011, Hanna 2012, Schouten 2012, Desmarchelier 2013, Tiusanen 2013, Vendittoli 2013, Zagra 2013, Engh 2014, Gustafson 2014, Zijlstra 2014, Ando 2015, Briggs 2015, Gofton 2015, Engh 2016, Dahlstrand 2017, Schouten 2017
Hip resurfacing	Vendittoli 2006, Girard 2006, Girard 2008, Lavigne 2010, Smolders 2010, Vendittoli 2010, Jensen 2011, Petersen 2011, Costa 2012, Penny 2012, Wang 2012, Penny 2013a, Lorenzen 2013, Gerhardt 2015, Tice 2015, Bisseling 2015, Costa 2018, Tao 2018, Gerhardt 2019.
Navigation	Kalteis 2006, Parratte 2007, Hart 2008, Sendtner 2011, Reininga 2013, Gurgel 2014, Lass 2014, Weber 2014, Renkawitz 2015, Parratte 2016, Sariali 2016, Weber 2016.
Technique	Bhan 2006, Flivik 2006, Vendittoli 2007, Rice 2014, Flivik 2015, Qu 2016, Rutherford 2019
Closure, drains, and postoperative care	Livesey 2009, Buttaro 2015, Walmsley 2005, Cheung 2010, Horstmann 2012, Kleinert 2012, Ström 2007a, Ström 2007b, Wolf 2010, Dietz 2019, Peters 2019, Thien 2007
